# Daytime eating prevents mood vulnerability in night work

**DOI:** 10.1073/pnas.2206348119

**Published:** 2022-09-12

**Authors:** Jingyi Qian, Nina Vujovic, Hoa Nguyen, Nishath Rahman, Su Wei Heng, Stephen Amira, Frank A. J. L. Scheer, Sarah L. Chellappa

**Affiliations:** ^a^Medical Chronobiology Program, Division of Sleep and Circadian Disorders, Departments of Medicine and Neurology, Brigham and Women's Hospital, Boston, MA, 02115;; ^b^Division of Sleep Medicine, Harvard Medical School, Boston, MA 02115;; ^c^Department of Nuclear Medicine, Faculty of Medicine, University Hospital Cologne, University of Cologne, Cologne, 50937, Germany

**Keywords:** mood vulnerability, circadian disruption, sleep, shift work, mental health

## Abstract

Shift workers have a 25 to 40% higher risk of depression and anxiety partly due to a misalignment between the central circadian clock and daily environmental/behavioral cycles that may negatively affect mood and emotional well-being. Hence, evidence-based circadian interventions are required to prevent mood vulnerability in shift work settings. We used a stringently controlled 14-d circadian paradigm to assess mood vulnerability during simulated night work with either daytime and nighttime or daytime-only eating as compared with simulated day work (baseline). Simulated night work with daytime and nighttime eating increased depression-like mood levels by 26.2% (*p*-value adjusted using False Discovery Rates, *p*FDR = 0.001; effect-size *r* = 0.78) and anxiety-like mood levels by 16.1% (*p*FDR = 0.001; effect-size *r* = 0.47) compared to baseline, whereas this did not occur with simulated night work in the daytime-only eating group. Importantly, a larger degree of internal circadian misalignment was robustly associated with more depression-like (*r* = 0.77; *P* = 0.001) and anxiety-like (*r* = 0.67; *P* = 0.002) mood levels during simulated night work. These findings offer a proof-of-concept demonstration of an evidence-based meal timing intervention that may prevent mood vulnerability in shift work settings. Future studies are required to establish if changes in meal timing can prevent mood vulnerability in night workers.

Shift workers have a 25 to 40% higher risk of depression and anxiety, with mental health disorders contributing to an estimated global cost of ∼US$1 trillion/y in lost workforce productivity alone (1,2). Shift workers often experience a misalignment between the central circadian clock and daily environmental/behavioral cycles, and circadian misalignment can negatively affect mood and emotional well-being in nonshift workers and shift workers (3–5). The identification of this important modifiable factor highlights the urgent need for evidence-based circadian interventions to prevent mood vulnerability in this at-risk population. Daytime eating—despite mistimed sleep—can maintain internal circadian alignment and prevent glucose intolerance during simulated night work (6). As impaired glycemic control is a risk factor for mood disruption (7), we tested the prediction that daytime eating prevents mood vulnerability, despite simulated night work. We performed a parallel-design randomized clinical trial (i.e., different participants randomly allocated to one of two meal timing groups) that included a 14-d circadian laboratory protocol with healthy participants (12 men, 7 women; age: 26.5 ± 4.1 y) (Fig. 1 *A* and *B*). Participants underwent a forced desynchrony (FD) protocol in dim light (∼3 lx) for 4 “days” of 28 h, such that each 28-h FD day resulted in an additional 4-h misalignment between the central circadian clock and external behavioral/environmental cycles. By the fourth FD day (simulated night work), participants were 12-h misaligned compared to the first FD day (baseline). In the daytime and nighttime meal control (DNMC) group, participants had a typical 28-h FD protocol, i.e., with behavioral and environmental cycles (sleep/wake, rest/activity, supine/upright posture, dark during scheduled sleep /dim light during wakefulness, etc.) scheduled on a 28-h cycle, including the fasting/eating cycle. Hence, meals occurred during both the daytime and nighttime, which is typical among night workers. In the daytime-only meal intervention (DMI) group, participants underwent a modified 28-h FD protocol with behavioral/environmental cycles scheduled on a 28-h cycle except for the fasting/eating cycle, which was scheduled on a 24-h cycle, thus resulting in meals consumed only during the daytime. We assessed depression-like and anxiety-like mood, which correspond to an amalgam of mood states typically observed in depression and anxiety, every hour during the 4 FD days using computerized visual analogue scales (VAS) (8) (*SI Appendix*).

**Fig. 1.**
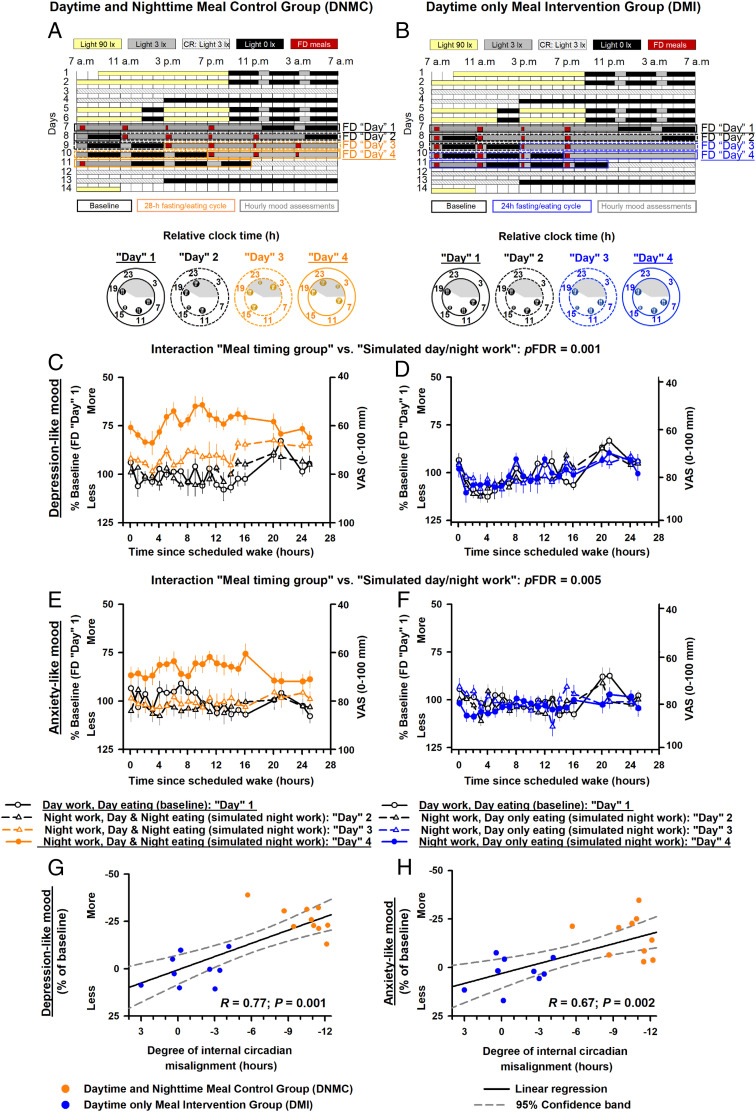
Experimental protocol (*A* and *B*), meal timing effects on depression-like mood (*C* and *D*) and on anxiety-like mood (*E* and *F*), and the association of depression-like mood (*G*) and of anxiety-like mood (*H*) with internal circadian misalignment (*SI Appendix*, *SI Text* has the degree of internal circadian misalignment estimation). VAS= Visual analogue scales.

## Results and Discussion

The meal timing intervention significantly modified the impact of simulated night work on depression-like mood levels (interaction “meal timing group” vs. “simulated day/night work”: *p*FDR *=* 0.001; effect-size *r* = 0.78). In the DNMC group, simulated night work increased depression-like mood levels by 26.2% relative to each participant’s baseline (95% confidence intervals, 21 to 31.5%; *P =* 0.001) (Fig. 1*C*). Conversely, in the DMI group, no significant effects were observed (−5.7 to 7.4%; *P =* not significant [n.s.]) (Fig. 1*D*). Similarly, the meal timing intervention significantly modified the impact of simulated night work on anxiety-like mood levels (interaction meal timing group vs. simulated day/night work: *p*FDR *=* 0.001; effect-size *r* = 0.47). In the DNMC group, simulated night work increased anxiety-like mood levels by 16.1% relative to each participant’s baseline (8.5 to 23.6%; *P =* 0.005) (Fig. 1*E*). Conversely, in the DMI group, no significant effects were observed (−3.1 to 9.9%; *P* = n.s.) (Fig. 1*F*). Importantly, we tested whether increased mood vulnerability during simulated night work was associated with the degree of internal circadian misalignment (i.e., change in the phase difference between the acrophase of circadian glucose rhythms and the bathyphase of circadian body temperature rhythms [expressed as the difference from baseline constant routine to postmisalignment constant routine protocol in hours]) (6). Accordingly, a larger degree of internal circadian misalignment was robustly associated with more depression-like and anxiety-like mood levels during simulated night work (linear regression models: *r* = 0.77; *P* = 0.001 and *r* = 0.67; *P* = 0.002, respectively) (Fig. 1 *G* and *H*).

We found evidence that meal timing had moderate to large effects on depression-like and anxiety-like mood levels during simulated night work, and that such effects were associated with the degree of internal circadian misalignment. These effects were unlikely due to differences in study design, as the laboratory protocol of both groups was identical (i.e., caloric/macronutrient intake, physical activity, posture, sleep duration, lighting conditions), *except for the timing of meals*. The relevance of diet on sleep, circadian rhythms and mental health is receiving growing awareness with the emergence of a new field, nutritional psychiatry (9, 10). A recent population-based cross-sectional study with 502,494 individuals showed an association of unhealthy diet with worse sleep quality and mental health symptomatology (11). Importantly, individuals experiencing depression often report poor-quality diets with high carbohydrate intake (9). In contrast, there is evidence for an association of (high adherence to) the Mediterranean diet with lower odds of depression, anxiety, and psychological distress (12, 13). While these emerging studies suggest an association between dietary factors and mental health, experimental studies in individuals with depressive and/or anxiety/anxiety-related disorders are required to determine causality and direction of effects. Meal timing is an emerging aspect of nutrition, with increasing research interest because of its influence on physical health (14). However, the causal role of the timing of food intake on mental health remains to be tested. Nighttime work with daytime and nighttime eating (using identical test meals with ∼50% carbohydrate intake) impaired glucose tolerance, whereas no such effects occurred in nighttime work with daytime-only eating (6). Therefore, the beneficial effects of daytime eating on glucose tolerance may extend to mood perception; however, future studies are required to establish the effects of meal timing on individuals experiencing depressive and/or anxiety/anxiety-related disorders (*SI Appendix* has additional discussion).

Hyperglycemia is a risk factor for depression (7). Support for this association comes from preclinical work that shows insulin receptor signaling as central for brain function and depression-like and anxiety-like behaviors (15). Accordingly, mice with neuronal-specific knockout of insulin receptors exhibit mitochondrial dysfunction, increased oxidative stress, and monoamine oxidase expression in brain regions classically involved in emotional processing, including the nucleus accumbens and amygdala (15). Importantly, this results in the occurence of depression-like and anxiety-like behaviors (15). Hence, by preventing glucose intolerance, daytime eating might also prevent mood vulnerability under shift work settings. Dietary factors may also affect mental health through its consequences on the gut microbiota, which is a critical hub for tryptophan/serotonin regulation, inflammation, oxidative stress, and neuroplasticity (9). Preclinical work shows that circadian disruption perturbs microbiota communities (16). Moreover, circadian misalignment in humans can alter microbiota composition, possibly with a shift toward proinflammatory taxa and decreased abundance in microbiota-mediated functional pathways, including tryptophan biosynthesis essential for serotonin production (17). Thus, circadian misalignment may adversely affect the microbiota with ramifications for mental health. In summary, our study offers proof of concept that daytime eating may prevent mood vulnerability in shift work schedules. Randomized controlled trials in shift workers will help establish if meal timing interventions can prevent depression and anxiety in real-world settings.

## Materials and Methods

The protocol was approved by the Partners HealthCare’s institutional review board and performed in accordance with the principles of the Declaration of Helsinki, and participants provided written informed consent. Detailed descriptions, protocol approval, and data availability are in *SI Appendix*.

## Supplementary Material

Supplementary File

## Data Availability

All study data are included in the article and/or *SI Appendix*.
