# Supplementary material for: Daytime eating prevents mood vulnerability in night work
Source: Proc Natl Acad Sci U S A. 2022 Sep 12;119(38):e2206348119. doi: 10.1073/pnas.2206348119 (PMC9499546; doi:10.1073/pnas.2206348119)
Supplement: Supplementary File [file pnas.2206348119.sapp.pdf]

PNAS

Supporting Information for
Daytime eating prevents mood vulnerability in night work

Jingyi Qian[a,b], Nina Vujovic [a,b], Hoa Nguyen[a], Nishath Rahman[a], Su Wei Heng[a], Stephen Amira[b], Frank A.J.L. Scheer[a,b,1], Sarah L. Chellappa[a,b,c,1,2]

[a] Medical Chronobiology Program, Division of Sleep and Circadian Disorders, Departments of Medicine and Neurology, Brigham and Women's Hospital, Boston, MA 02115, United States.

[b] Division of Sleep Medicine, Harvard Medical School, Boston, MA 02115, United States.

[c] Department of Nuclear Medicine, Faculty of Medicine and University Hospital Cologne, University of Cologne, Cologne 50937, Germany.

[1] To whom correspondence may be addressed. Email: fscheer@bwh.harvard.edu or sarah.chellappa@outlook.com.

[2] F.A.J.L.S. and S.L.C. contributed equally to this work.

Supporting Information Text

Data availability

All data needed to evaluate the conclusions in the paper are present in the paper (main text and SI appendix). As per the NIH Policy on Data Sharing, we will make the datasets available to other investigators following publication of the final study results. These datasets will not contain identifying information per the regulations outlined in HIPPA. Per standard Partners HealthCare System policies, we will require from any investigator or entity requesting the data a data-sharing agreement that provides for the following. (i) a commitment to using the data only for research purposes and not to identify any individual participant, (ii) a commitment to securing the data using appropriate computer technology, and (iii) a commitment to destroying or returning the data after analyses are completed.

Participants

Participants admitted to the laboratory protocols were free from medical conditions, including current and previous history for e.g., depressive and anxiety/anxiety-related disorders. Medical suitability was assessed using clinical history, biochemical and toxicology blood and urine screenings, and physical and psychological exams. Participants completed the Minnesota Multiphasic Personality Inventory (MMPI-2) psychometric clinical scale during screening. Participants were non-smokers and not taking caffeine, drugs or any medications (except oral contraceptives), as assessed by a urine toxicological screening. Participants underwent a randomized, parallel, controlled, single-blinded trial (1), in which they were randomly assigned to one of two meal timing groups. The Daytime and Nighttime Meal Control Group (DNMC) completed a protocol that included simulated day work with day eating (baseline) followed by simulated night work with daytime and nighttime eating, typical in shift workers. The Daytime only Meal Intervention

Group (DMI) completed a protocol that included baseline followed by simulated night work with daytime-eating only.

Twenty participants (mean age, 26.6y [Standard deviation, SD, 4.2y, range: 18-35y], eight women, BMI range: 18.5-29.9kg/m^2, hemoglobin A1C range: 4.9-5.4%) were randomized to the laboratory protocol: ten participants were allocated to the DNMC Group and ten to the DMI Group (**Figures 1A,B**). Four women commenced the laboratory protocol on menstrual cycle days 1-5 (two per meal timing group) and four during days 14-19 (two per meal timing group). We excluded data from one participant in the DMI Group due to their inability to consume all meals during the simulated night work. The final study sample included ten participants for the DNMC Group (mean age, 27.0 years [SD, 4.4y], 4 women, BMI: 22.5 kg/m^2 [SD, 3.5]), and nine for the DMI Group (mean age, 26.2 years [SD, 4.1y], 3 women, BMI: 23.1 kg/m^2 [SD, 3.1]). Additional study-related characteristics between groups (e.g., diet, chronotype, among others) are described in (1). No statistical differences in participant demographics and study-related characteristics occurred between groups.

Study design

Before the laboratory protocol, participants maintained a fixed, self-selected habitual bedtime with 8-h time in bed for ~2 weeks. We verified compliance with ambulatory actigraphy (Actiwatch, Respironics), sleep logs, and time-stamped voicemails. During the three days before the laboratory protocol, participants received all meals (three meals and one snack) from the Metabolic Kitchen to meet dietary requirements (Harris Benedict formula with activity factor 1.4) and controlled macronutrient distribution (45-50% carbohydrate, 15-20% protein, 30-35% fat, with 150 mEq Na+ (± 20%), 100 mEq K+ (±20%), which matched the subsequent laboratory diet. Participants were requested to consume the provided meals at their habitual eating times to standardize the amount, type and timing of food intake before the laboratory protocol. The latter was ensured by participants calling into a time-stamped voice-mailbox when they began each meal and by a daily food log that included food content and times.

During the laboratory protocol, participants remained in individual laboratory suites in an environment free of time cues. Throughout the study, when participants were not involved in a study task, they could undertake leisure activities, such as reading, writing, watching movies, crafts, etc. We monitored each participant's activity for compliance with closed circuit TV and wrist-worn actigraphy.

Days 1-2 comprised the laboratory adaptation days. Days 3-4 were a baseline constant routine (CR) protocol (**Figures 1A,B**), during which participants spent 32-h continuously awake in a constant semi-recumbent body posture, without physical exertion, in dim light (~3 lx in the horizontal angle of gaze) and eating hourly isocaloric snacks. This allowed a baseline assessment of endogenous circadian rhythms of metabolic markers and core body temperature (1) by minimizing the influences of behavioral and environmental factors on a given rhythm (2). Following the baseline CR, participants had a 12-h sleep opportunity to recover. On Days 5 and 6, participants had further recovery from the baseline CR. On Day 7, participants underwent a 28-h Forced Desynchrony protocol (FD) to induce circadian misalignment, with 28-h sleep/wake cycles under dim light (~3 lx) during wakefulness (and no light during sleep), to which the central circadian pacemaker in humans cannot entrain (3). We used a 28-h FD protocol to assess the impact of circadian misalignment on metabolic function (4). During each 28-h cycle, the ratio of scheduled wakefulness (18h:40min) and sleep (9h:20min) was maintained at 2:1, to match the self-selected 8 h habitual time in bed per 24 h. The participants' sleep episodes were split into three identical blocks, each separated by 1 h of wakefulness in dim light while remaining at rest in a semi-recumbent posture in bed. This allowed the participants to consume food during the circadian day when otherwise they would be sleeping. Importantly, participants woke during each sleep episode irrespective of meal consumption to ensure that both study groups had three equal sleep blocks during the FD protocol. On the first 28-h sleep/wake cycle, participants had normal circadian alignment (waking up at their habitual wake-up time, e.g., 7a.m.; baseline). Participants underwent the FD protocol for 4 "days" of 28 h, such that each FD day resulted in an additional 4-h misalignment between the central circadian phase and external behavioral/environmental cycles. Therefore, by the fourth sleep/wake cycle, participants were 12-h misaligned (wake-up time, e.g.,

7p.m.; simulated night work) as compared to the first cycle in both groups. The DNMC Group (**Figure 1A**) was a traditional 28-h FD protocol with all behaviors, including the fasting/eating cycle, maintained on a 28-h cycle. Because of that, the three meals and a snack were scheduled at fixed times relative to scheduled wake-up time (i.e., at 0h:10min, 4h:10min, 8h:10min, and 12h:10min since scheduled awakening, during baseline and simulated night work). Thus, each meal was shifted 4 h later each cycle, in alignment with the sleep/wake cycle. Participants thus consumed food during both the daytime and nighttime, which is typical behavior of night shift workers. In contrast, the DMI Group (**Figure 1B**) was a modified 28-h FD protocol, with all behaviors identically scheduled on a 28-h cycle, except for the fasting/eating cycle that was maintained on a 24-h cycle. Accordingly, participants had standardized meals at 0h:10min, 4h:10min, 8h:10min, and 12h:10min since scheduled awakening during baseline, whereas meals occurred at 0h:10min, 12h:10min, 16h:10min, and 20h:10min since scheduled awakening during the simulated night work. This approach allowed alignment of the fasting/eating cycle to the ~24-h central circadian cycle and ensured meal consumption only during the daytime and at the same clock time during each FD cycle. Subsequent to the four "days" of a 28-h FD protocol, participants underwent a post-misalignment CR (Days 11-13) that allowed assessing the impact of prior circadian misalignment on endogenous central and peripheral circadian rhythms. During the post-misalignment CR, participants spent 40-h under the same experimental conditions described for the baseline CR. Thereafter, participants were scheduled to a 12-h sleep opportunity to allow them to recover (partially) from the post-misalignment CR protocol, and then were discharged from the study. Details of the diet are provided in (1).

Mood vulnerability measurements

Circadian rhythm disruption is robustly associated with depression and with anxiety (5). To determine whether circadian misalignment affects depression-like and anxiety-like mood and if a daytime meal intervention can prevent such adverse mood effects, we assessed a plethora of mood perception measures every hour during the four FD "days" using computerized visual analogue scales (VAS). The VAS probes affective multidimensional components encompassing depression-like and anxiety-like mood (6). The VAS is a 100-mm horizontal line presented during computerized test sessions with a word at each end that represents the extremes of, e.g., affective perception, for example, between "Sad" and "Happy". Participants were requested to place a mark at that point on the line that represents their current emotional state along that continuum. In each computerized test session, sixteen affective components were assessed: "Sad/Happy", "Hostile/Friendly", "Bored/Interested", "Withdrawn/Sociable", "Excited/Calm", "Troubled/Tranquil", "Discontented/Contended"", "Tense/Relaxed", "Groggy/Clearheaded", "Mentally slow/Quick-witted", "Dreamy/Attentive", "Incompetent/Competent", "Weak/Strong", "Clumsy/Well coordinated", "Sluggish/Energetic", and "Cold/Warm". Depression-like mood was defined as a composite score averaged over four items: "Sad/Happy", "Hostile/Friendly", "Bored/Interested" and "Withdrawn/Sociable" (6-8). Anxiety-like mood was defined as a composite score averaged over four items: "Excited/Calm", "Troubled/Tranquil", "Discontented/Contended" and "Tense/Relaxed" (6-8). "Depression-like" and "Anxiety-like" mood thus correspond to the amalgam of mood states typically observed in depressive disorders and anxiety/anxiety-related disorders. Mental fatigue was defined as a composite score averaged over four items: "Groggy/Clearheaded", "Mentally slow/Quick-witted", "Dreamy/Attentive" and "Incompetent/Competent" [6-8]. Physical comfort was defined as a composite score averaged over four items: "Weak/Strong", "Clumsy/ Well coordinated", "Sluggish/Energetic", and "Cold/Warm" (6-8). These VAS-derived mood state measurements have been extensively validated in previous laboratory studies (8-12) and can be reliably used for sequential serial time measurements due to the absence of ceiling effects. We did not use the PANAS or the POMS, which allow for the multidimensional assessment of mood (e.g., arousal and valence), because these affective scales may not be ideally suited for serial time measurements (single measurements are most typical when using these scales, for which they were originally designed).

Sleep measurements

We assessed the influence of prior sleep on mood vulnerability (i.e., depression-like and anxiety-like mood) before the baseline and simulated night work. Sleep was measured using electroencephalographic (EEG), electrooculogram (EOG), and submental electromyography (EMG) using American Academy of Sleep Medicine recommended EEG derivations (C3/4, F3/4, O1/2, referenced to M2/1) (Vitaport recorder, TEMEC, Netherlands). Sleep structure included wake during sleep, non-rapid eye movement (NREM) sleep stages 1-3, rapid eye movement (REM) sleep, sleep efficiency and sleep onset latency.

Quantification of internal circadian misalignment

The degree of internal circadian misalignment was indexed by the change in the circadian phase difference between the endogenous circadian glucose rhythms and the endogenous circadian core body temperature (CBT) rhythms from Baseline CR to Post-misalignment CR in hours. Endogenous circadian glucose rhythms, which were a proxy for peripheral circadian oscillations, were determined from plasma glucose measurements obtained through hourly blood samples during the Baseline and Post-misalignment CR protocols. Endogenous circadian CBT rhythms, which were a proxy for central circadian oscillations, were continuously measured during both CR protocols using a flexible rectal temperature sensor (Yellow Springs Instrument Company, OH, USA), and assessed CBT every 1 min. To determine the circadian phase of the endogenous circadian glucose and CBT rhythms, we applied cosinor mixed model analyses [see (1) for more extensive methods regarding glucose and CBT endpoints].

Quantification of glucose tolerance during baseline and simulated night work

To assess glucose tolerance, four identical Test meals per participant were strictly timed at 0h:10-min and 12h:10min since scheduled wakefulness (thus 12 h apart) on the days of baseline and simulated night work (during the FD protocol). Test meals occurred once during the morning (e.g., 7:10a.m., for a participant with a habitual wake-up time of 7a.m.) and once during the evening (e.g., 7:10p.m., for a participant with a habitual wake-up time of 7a.m.) per baseline and per simulated night work (for details, see (1)). Measurements of plasma glucose happened before each Test meal (fasting blood was drawn ~7 min before each Test meal), and thereafter every 10 min for 90 min, then every 30 min for another 90 min. Here, glucose tolerance was indexed as the average 3-h postprandial glucose profile after each Test meal per baseline and per simulated night work. Of note, in the DNMC and DMI Groups, fasting duration prior to the first meal during baseline was ~12 h and ~4 h prior to the second, third, and fourth meal/snack. In the DNMC Group, fasting duration prior to the first meal (breakfast) during the simulated night work was ~16 h for the first and ~4 h prior to the other meals/snack. In the DMI Group, fasting duration prior to the second meal (breakfast) during the simulated night work was ~12 h and ~4 h prior to the other meals/snack. This fasting duration of ~12 h or ~16 h before breakfast is in accordance with the recommended fasting duration for clinical glucose tolerance tests by the American Diabetes Association, which recommends overnight fasting for at least 8 h, with varying durations of 8-16 h (13,14). Hence, the differences in fasting duration prior to the Test meals are unlikely to have mediated the effects of meal timing on glucose tolerance.

Statistical analysis

We performed statistical analyses using SAS version 9.4 (SAS Institute, Cary, NC, USA) and SigmaPlot version 14.0 for the linear regressions and illustrations. The sample size was derived from the expected difference in the effect of misalignment on glucose tolerance (3-h postprandial plasma glucose profiles) between the meal timing groups. To determine a large effect size (d=1.5) with ~80% power, eight participants per group were required (total sample=16). We increased the number of participants per group to 10 to mitigate potential data losses. We

compared participants' characteristics with Yates's chi-squared tests or *t*-tests for independent groups, and their demographics and study-related characteristics did not significantly differ between the meal timing groups.

The depression-like and anxiety-like mood outcomes were normalized by using an average of each participant's levels measured throughout baseline (i.e., simulated day work; 1st FD "day") to minimize any effect of interindividual differences in baseline mood perception. Of note, parallel study designs in particular require data normalization. Mixed-model analyses of variance included three main factors: 1) Meal timing group (DNMC Group vs. DMI Group); 2) Simulated day/night work (baseline [simulated day work] vs. simulated night work); 3) Time (hours into each simulated day and night shift cycles) (reported in **Figures 1C-F**). The interaction of meal timing group and simulated day/night work was used to identify whether the meal timing intervention significantly modified the impact of simulated night work on depression-like and anxiety-like mood levels during the four FD "days". Post-hoc comparisons used the Tukey-Kramer test to adjust for multiple testing. Participant was included as a random factor.

Sleep can influence mood vulnerability [10], hence, we assessed sleep structure characteristics before the baseline and simulated night work conditions in both meal timing groups using mixed-model analyses of variance with main factors Meal timing group and Simulated day/night work, as well as their interaction. Accordingly, we observed no significant effects [see (1) for details]. Additionally, we calculated the effect sizes of the meal timing intervention on depression-like and anxiety-like mood by assessing the effect-size correlation, where r was computed using the means and standard deviations of two independent groups [intervention (DMI Group) and control (DNMC Group)]. Because the ability to sleep (e.g., sleep efficiency) has been shown to strongly affect mood vulnerability (10), we tested whether sleep efficiency before the baseline and simulated night work affected the reported effects on depression-like and anxiety-like mood levels in both meal timing groups. For the sleep analyses, we included the three sleep episodes before baseline (simulated day work) and the three sleep episodes before the simulated night work into a single sleep episode, and thereof estimated sleep efficiency (1). We thus performed the abovementioned mixed-model analyses of variance here including sleep efficiency as covariate of interest as in (10). Additionally, we performed linear regression models to assess whether there were associations between the magnitudes of internal circadian misalignment (change from baseline CR to post-misalignment CR) with the mean 28-h depression-like and with the mean 28-h anxiety-like mood levels during circadian misalignment (change from baseline to simulated night work, **Figures 1G,H**).

To control overall type I error in null hypothesis testing when conducting multiple comparisons, *P*-values from the mixed-model analysis were adjusted using False Discovery Rates (*p*FDR). Data presented in the main text correspond to the median and the 95% confidence intervals (i.e., absolute difference from baseline to simulated night work). Data presented in Figures 1C-F are the mean and standard error of mean. Significance for statistical tests was set as $P < 0.05$.

SI Results and Discussion

We found evidence that meal timing had moderate to large effects on depression-like and anxiety-like mood levels during simulated night work, and that such effects were associated with the degree of internal circadian misalignment. Our reported effects did not stem from differences in participant demographics, study-related/psychometric characteristics and sleep structure (all $p =$ non-significant, n.s.). Because sleep influences mood vulnerability (10), we tested whether the ability to sleep (sleep efficiency) significantly affected depression-like and anxiety-like mood levels. Accordingly, the reported meal timing effects remained significant even when including sleep efficiency as a covariate (interaction "Meal timing group" vs. "Simulated day/night work": p=0.001, p=0.002, respectively). Likewise, no group differences occurred for mental fatigue, physical comfort and subjective sleepiness (all P = n.s.). Nighttime work with daytime and nighttime eating can impair glucose tolerance, whereas this is not observed during simulated night work with daytime-only eating (1). Hence, the beneficial effects of daytime only eating on glucose tolerance may likely extend to mood perception. Indeed, glucose intolerance induced by internal circadian misalignment

was associated with more depression-like and anxiety-like mood levels during simulated night work (linear regression models: respectively, $r = 0.57$; $P = 0.01$; $r = 0.47$; $P = 0.03$). These findings reinforce the assumption that daytime only eating—by preventing glucose intolerance—may also prevent mood vulnerability despite mistimed sleep. Importantly, our reported effects were observed following four days under the 28-h FD protocol, during which participants in both meal timing groups experienced a misalignment by 12 h of most behavioral/environmental cycles (except for the fasting/eating cycle in the DMI group) with the central circadian clock. We previously assessed the effects of circadian misalignment on mood vulnerability in a laboratory study whereby behavioral and environmental cycles (including sleep/wake, fasting/eating, rest/activity, dark/light) were aligned (circadian alignment) or misaligned (after a rapid 12 h shift of environmental/behavioral cycles) with the central circadian clock (10). Accordingly, we observed that the 12 h shift of the environmental/behavioral cycles relative to the central circadian clock could adversely affect mood states. In our current study, we show similar effects during FD "day" 4 (with a similar misalignment by 12 h) in the DNMC group, while "days" 2-3 had a smaller degree of misalignment (4-8 h). Such findings suggest that a shift of the fasting/eating cycle relative to the central circadian clock - as typically experienced in night shift work - might underlie the reported adverse mood effects of circadian misalignment. Preclinical work has shown that mistimed feeding causes phase shifts of the expression rhythm of clock genes in the hippocampus, and might change the expression levels of monoamines thereby, leading to malfunction of serotonin and norepinephrine in the hippocampus and more depression-like behavior (15). As such, it is likely that mistimed eating has a critical role in mood regulation.

Here, we assessed the VAS mood constructs in healthy individuals and not in patients experiencing depressive disorders and/or anxiety/anxiety-related disorders. Participants had Multiphasic Personality Inventory (MMPI-2) subscale values within average typical scores for i.e., hypochondriasis, depression, hysteria, psychopathic deviate, paranoia, psychasthenia, schizophrenia, hypomania, and social introversion. Importantly, we observed no statistically significant differences for any of the MMPI-2 subscales between the two meal timing groups (t-tests for independent groups, all P = n.s.). As such, participants were deemed as not having current and/or a history of depressive disorders and anxiety/anxiety-related disorders. Our current findings therefore required future studies to determine if such meal timing effects translate to patient populations.

To test for the effects of circadian misalignment on mood vulnerability and whether appropriate meal timing could prevent the potential adverse effects of circadian misalignment on mood perception, our laboratory protocol was stringently controlled for a myriad of potential confounders, including the impact of environmental and social cues. Human studies indicate that when individuals have knowledge of others' behavior in the same social setting, they are more likely to make similar selections or consume similar amounts of food (16). Potential underlying factors motivating such eating behaviors come from preclinical work in which environmental/social cues have been shown influence homeostatic and hedonic brain signals, which, in turn, may lead to increases in the affective taste properties of food and desire to consume it (17). These findings suggest that the lack of environmental cues might influence food consumption. Randomized controlled trials including individuals engaged in shift work are therefore required to test whether environment cues might affect our reported effects of meal timing on mood perception. In similar vein, physical activity was restricted in the laboratory protocol to minimize its effects on glucose tolerance (1). While physical inactivity may reduce mood levels in shift work settings, this association depends on a myriad of factors, including age, gender, years of exposure and type of shift work (18,19). Moreover, shift workers tend to be less physically active as compared to non-shift working counterparts, partly because of demanding work schedules, reduced access to exercise facilities and/or circadian rhythm disruption (20). As such - even if physical activity is beneficial to glucose homeostasis and mood levels - shift workers might be less likely to benefit from it as compared to non-shift workers. Collectively, it is challenging to predict how physical action/movement may affect mood under different shift work settings. Importantly, we assessed mood perception throughout the FD protocol and if there were a depressogenic and/or anxiogenic effect about restricted movement, we would expect substantial differences between the first and second FD "days". That is because participants were not in semi-recumbent posture during the scheduled wake episode on FD "day" 2, whereas on FD "day" 1 the participants had a mix of semi-

recumbent posture after the two Test meals and could move during the remainder of the wake episode of FD “day”1. As illustrated on Figures 1C-D and 1E-F, there were no substantial differences during FD “days” 1 and 2 for depression-like and anxiety-like mood levels in either meal timing group. Additionally, the scheduled postural changes throughout the FD protocols were identical between the DNMC and DMI groups. Therefore, it is unlikely that changes in posture account for the observed differences between the two groups. Furthermore, participants did not have depressive disorders and/or anxiety/anxiety-related disorders, and their better mental health status might have buffered the effect that restricted movement might induce in individuals experiencing a mental illness. Of note, sleep/circadian laboratory protocols are inherently challenging. As such, stringent psychological screening procedures, inclusion/exclusion criteria on standard questionnaires and clinical interview questions are conducted to ensure continued psychological health during such laboratory protocols (21).

A limitation in our study is the restricted age range of the study participants. Healthy older individuals may experience lower subjective mood and well-being levels as compared to younger individuals (12). This could be to a decrease in subjective mood perception or diminishing physiological and/or psychological constitution. Moreover, healthy older individuals may experience more impaired subjective mood and well-being under sleep deprivation than younger individuals (12). This may imply a greater vulnerability to sleep loss with age. As night shift workers often experience sleep restriction/loss due to their work schedules, older individuals who engage in night work might experience increased mood vulnerability as compared to younger individuals. However, as there are no studies on meal timing effects, aging and mood vulnerability, it remains to be tested whether our reported beneficial effects of meal timing on mood perception extend to older individuals. Importantly, our proof-of-concept demonstration of an evidence-based meal timing intervention that may prevent mood vulnerability in shift work schedules requires replication in real-life settings. While laboratory approaches offer greatest experimental control, its findings have limited direct translation to night shift workers, as they do not live under such carefully controlled behavioral and environmental conditions. Moreover, a field-based approach can allow for real-time mobile monitoring of the multiple associations among motor activity, mood and sleep in individuals experiencing mood disorders (22). Hence, naturalistic studies with larger samples (23) including night shift workers and that track the timing of food intake, sleep and mood will help establish if changes in meal timing can be applied to reduce depressed and anxious mood states in night shift workers.

In summary, our reported findings show that daytime only eating (despite mistimed sleep) may minimize the adverse effects of circadian misalignment on depression-like and anxiety-like mood levels. These data offer evidence for meal timing interventions that may potentially help in mental illness prevention among shift work populations and other individuals experiencing circadian rhythm disturbances.

Related articles

Other aspects of these laboratory protocols, designed to test independent hypotheses, have been published previously (1,24).

SI References

1. S. L. Chellappa et al., Daytime eating prevents internal circadian misalignment and glucose intolerance in night work. Sci Adv 7, eabg9910 (2021).
2. J. F. Duffy, D. J. Dijk, Getting through to circadian oscillators: why use constant routines? J Biol Rhythms 17, 4-13 (2002).
3. C. A. Czeisler et al., Stability, precision, and near-24-hour period of the human circadian pacemaker. Science 284, 2177-2181 (1999).
4. F. A. Scheer, M. F. Hilton, C. S. Mantzoros, S. A. Shea, Adverse metabolic and cardiovascular consequences of circadian misalignment. Proc Natl Acad Sci U S A 106, 4453-4458 (2009).

5. W. H. Walker, 2nd, J. C. Walton, A. C. DeVries, R. J. Nelson, Circadian rhythm disruption and mental health. Transl Psychiatry 10, 28 (2020).
6. A. Bond, M. Lader, The use of analogue scales in rating subjective feelings. Br J Med Psychol 47, 211-218 (1974).
7. R. C. B. Aitken, Measurement of feelings using visual analogue scales. . Proc R Soc Med 62, 989-993 (1969).
8. T. H. Monk, A Visual Analogue Scale technique to measure global vigor and affect. Psychiatry Res 27, 89-99 (1989).
9. P. Boudreau, G. A. Dumont, D. B. Boivin, Circadian adaptation to night shift work influences sleep, performance, mood and the autonomic modulation of the heart. PLoS One 8, e70813 (2013).
10. S. L. Chellappa, C. J. Morris, F. A. J. L. Scheer, Circadian misalignment increases mood vulnerability in simulated shift work. Sci Rep 10, 18614 (2020).
11. A. Birchler-Pedross et al., Subjective Mood in Young Unmedicated Depressed Women under High and Low Sleep Pressure Conditions. Biology (Basel) 5 (2016).
12. A. Birchler-Pedross et al., Subjective well-being is modulated by circadian phase, sleep pressure, age, and gender. J Biol Rhythms 24, 232-242 (2009).
13. A. American Diabetes, 2. Classification and Diagnosis of Diabetes: Standards of Medical Care in Diabetes-2020. Diabetes Care 43, S14-S31 (2020).
14. D. Bogdanet, P. O'Shea, C. Lyons, A. Shafat, F. Dunne, The Oral Glucose Tolerance Test-Is It Time for a Change?-A Literature Review with an Emphasis on Pregnancy. J Clin Med 9, (2020).
15. Haraguchi et al., Night eating model shows time-specific depression-like behavior in the forced swimming test. Sci Rep 8: 1081 (2018)
16. S. Prinsen et al., Eating by example. Effects of environmental cues on dietary decisions. Appetite 70:1-5 (2013).
17. A. W. Johnson. Eating beyond metabolic need: how environmental cues influence feeding behavior. Trends Neurosci 36(2):101-9 (2013).
18. T. Feng et al., A multimodal analysis of physical activity, sleep, and work shift in nurses with wearable sensor data. Sci Rep 11(1):8693 (2021).
19. M. Monnaatsie et al., Physical activity and sedentary behaviour in shift and non-shift workers: A systematic review and meta-analysis. Prev Med Rep;24:101597 (2021).
20. G. Atkinson et al., Exercise, energy balance and the shift worker. Sports Med;38(8):671-85 (2008).
21. S. Amira et al., Psychological Screening for Exceptional Environments: Laboratory Circadian Rhythm and Sleep Research. Clocks Sleep;2(2):13 (2020).
22. K. R. Merikangas et al., Real-time mobile monitoring of the dynamic associations among motor activity, energy, mood, and sleep in adults with bipolar disorder. JAMA Psychiatry; 76(2):190-198 (2019).
23. Wellcome Trust, 2022. Sleep, circadian rhythms and mental health: Advances, gaps, challenges and opportunities. Public-use data file and documentation. Available as a download PDF from: https://wellcome.org/reports/sleep-circadian-rhythms-and-mental-health-advances-gaps-challenges-and-opportunities.
24. S. L. Chellappa et al., Proof-of-principle demonstration of endogenous circadian system and circadian misalignment effects on human oral microbiota. FASEB J; 36(1):e22043.
